# Ligand Strategies for Regulating Atomically Precise CeO_2_ Nanoparticles: From Structure to Property

**DOI:** 10.3390/molecules30040846

**Published:** 2025-02-12

**Authors:** Peiling Du, Simin Li, Qinghua Xu, Ayisha He, Wei Yuan, Xinping Qu, Baimei Tan, Xinhuan Niu, Fan Zhang, Hui Shen

**Affiliations:** 1 College of Energy Materials and Chemistry, Inner Mongolia University, Hohhot 010021, China; pld202202@163.com (P.D.); siminli@imu.edu.cn (S.L.); xqh20202022@163.com (Q.X.); ayisha0218@163.com (A.H.); 2 Shanghai Frontiers Science Research Base of Intelligent Optoelectronics and Perception, Institute of Optoelectronics, Fudan University, 2005 Songhu Road, Shanghai 200438, China; yuanwei@fudan.edu.cn; 3 School of Microelectronics, Fudan University, Shanghai 200433, China; xpqu@fudan.edu.cn; 4 School of Electronics and Information Engineering, Hebei University of Technology, Tianjin 300130, China; bmtan@hebut.edu.cn (B.T.); xhniu@hebut.edu.cn (X.N.)

**Keywords:** ceria, nanocluster, structure, photoelectric conversion

## Abstract

The increasing interest in studying the structure–property relationships of ceria dioxide (CeO_2_) relies on the fact that many factors are key to determining the performance of CeO_2_ materials. Despite the great advances achieved, it remains a formidable challenge to regulate CeO_2_ nanoparticles at the molecular level and gain in-depth insight into their structure–property relationships. What is reported here is a ligand strategy for regulating CeO_2_ nanoparticles, in terms of not only shape, structure, surface composition, but also property. Atomically precise CeO_2_ nanoparticles (also named nanoclusters) are used as a model system, in which two Ce_16_ clusters are gained by a wet-chemical synthesis method. Featuring different carboxylate ligands on the surface, the two clusters are distinct in formula, core geometry, surface composition, and photoelectric merits. This work not only reports the first pair of atomically precise CeO_2_ nanoclusters with the same number of Ce atoms but different structures, which is highly desirable for studying structure–property relationships, but also provides in-depth insight into the molecular ligand effect in CeO_2_ materials.

## 1. Introduction

Featuring unique geometric structures and electronic properties (e.g., excellent redox ability, stable chemical properties), ceria dioxide (CeO_2_) has received ever-increasing attention in material science [1,2]. The nanoparticulate CeO_2_ has been enthusiastically employed in various practical areas such as chemical–mechanical polishing (CMP), catalysis (often as additives for heterogenous catalysts), ultraviolet absorption, solar energy conversion, and fuel cells [3,4,5,6,7,8,9,10,11]. It has been well known that the properties of CeO_2_ nanomaterials are highly sensitive to their structures including size, shape, and surface composition (especially Ce^3+^/Ce^4+^ ratios) [12]. In this context, regulating the structure of CeO_2_ nanoparticles, ideally at the molecular level, is urgently demanded to gain atomistic insight into the structure–property relationships of CeO_2_-containing materials [13,14].

The success in studying metallic nanoparticles (particularly coinage metals of Au, Ag, and Cu) by “atomically precise nanochemistry” provides a reference for studying the structure–property relationships of other nanomaterials [15,16,17,18,19]. On one hand, the size, shape, and composition of atomically precise nanoparticles (also called nanoclusters) are well defined and in some cases their molecule structures are determined by X-ray single-crystal diffraction analysis [20,21,22,23,24,25,26,27,28,29,30,31,32,33,34]. On the other hand, the optical, electronic, and catalytic properties of atomically precise metal nanoclusters are heavily dependent upon their structures, offering great potential to regulate performance at the molecular level [35,36,37,38,39,40,41,42,43,44,45,46,47,48,49,50]. Nevertheless, in sharp contrast to the rich chemistry of the atomically precise nanoparticles of coinage metals, parallel studies on CeO_2_ remain rather limited [38,51,52,53,54,55,56]. Recently, George Christou’s group reported the isolation and molecular structure of several atomically precise nanoparticles of CeO_2_, which provided the possibility to gain insight into the structure–property relationships of CeO_2_ materials. Some highlighted examples are [Ce_6_O_4_(OH)_4_(dmb)_12_(H_2_O)_4_] (dmb = 2,6-dimethoxybenzoate), Ce_16_O_17_(OH)_6_(O_2_CPh)_24_(HO_2_CPh)_4_, Ce_24_O_28_(OH)_8_(O_2_CPh)_30_(py)_4_, Ce_38_O_54_(OH)_8_(O_2_CEt)_36_(py)_8_ (py = pyridine), Ce_40_O_54_(OH)_4_(O_2_CMe)_46_(py)_4_, and [Ce_100_O_149_(OH)_18_(O_2_CPh)_60_(PhCO_2_H)_12_(H_2_O)_20_]^16+^ [57,58,59]. Although the molecular structures of several atomically precise CeO_2_ nanoparticles have been illustrated in previous reports [58,60], they raised open questions on how to regulate their structures and how differences in structure play a role in their properties.

In this work, we report a ligand strategy, for the first time, for regulating both the structures and properties of CeO_2_ nanoparticles. By using two kinds of carboxylate acids that are different in steric hinderance (benzoic acid and pivalic acid in this case) as surface modifiers, two atomically precise Ce_16_ clusters ([Ce_16_O_16_(OH)_8_(O_2_CPh)_20_(py)_8_(NO_3_)_2_] and [Ce_16_O_17_(OH)_6_(O_2_C^t^Bu)_24_(py)], labeled as **1** and **2**, respectively) were gained simply. The clusters share the same number of metal ions but have totally different geometric, electronic, and surface structures. More importantly, they exhibit distinct photoelectric properties, thus showcasing the significance of molecular structure in regulating the properties of CeO_2_ materials.

## 2. Results and Discussion

The carboxylate approach toward the modulation of the structures and properties of atomically precise CeO_2_ nanoparticles was inspired by the observation of a significant “carboxylate effect” in determining the properties of copper nanoclusters [61,62,63]. In our recent work, it has been revealed that the optical, catalytic, and electronic merits of copper nanoclusters are highly sensitive to the carboxylate ligands. It thus occurred to us that such a strategy might be applicable to regulating structured CeO_2_ nanoparticles.

Synthetically, the Ce_16_ clusters in this work were prepared by using the prototype reported by Mitchell et al.; namely, compounds **1** and **2** were obtained from the reaction of (NH_4_)_2_[Ce(NO_3_)_6_], pyridine, PhCO_2_H, or C(CH_3_)_3_COOH, respectively [58]. In a typical synthesis of cluster **1**, (NH_4_)_2_[Ce(NO_3_)_6_] was first suspended in a solvent of pyridine, followed by the addition of PhCO_2_H and CH_3_OH. The mixture was aged at room temperature by stirring for 30 min, followed by filtration. A clear yellow solution was obtained, which was layered with acetonitrile solution. The sample was kept undisturbed in a dark environment for 2 weeks, and yellow crystals were finally obtained with a yield of 4.4%, with some unknown powder precipitated (Figure S1). We note that the addition of methanol is rather important for the formation of **1**, as other discrete compounds (such as Ce_24_O_28_(OH)_8_(O_2_CPh)_30_(py)_4_) were always obtained without the participation of methanol. The function of methanol in the synthesis is proposed to affect the hydrolysis process and thus the resultant products. Cluster **2** was synthesized by using trimethylacetic acid under the same reaction conditions, affording bright yellow crystals with a yield of 4.7% along with some unknown powdered precipitation. Some key parameters of cluster synthesis are listed in Table 1.

**Figure 1:**
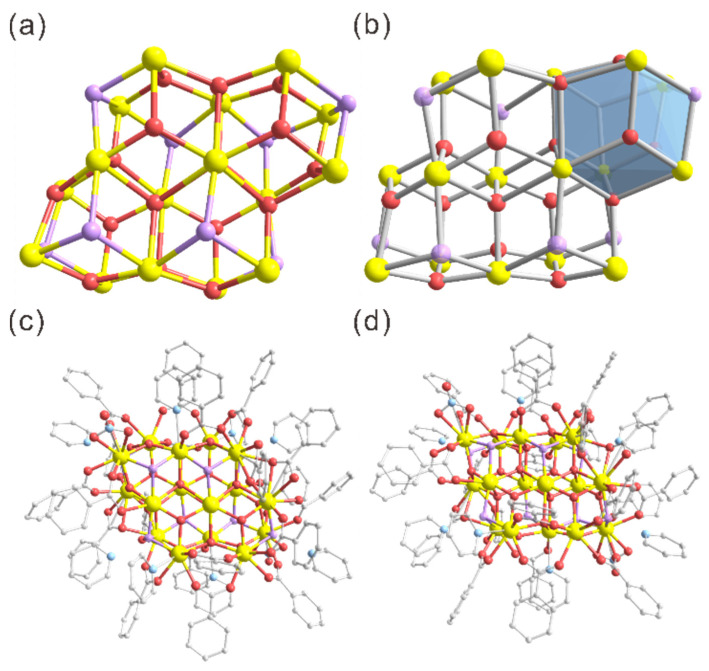
Structural anatomy of **1**. (**a**) {Ce_16_O_16_(OH)_8_} core and (**b**) secondary building units (blue pseudo dodecahedra) of **1**; (**c**,**d**) total structure of **1** from a-axis (**c**) and b-axis direction view (**d**). Color codes for atoms: yellow spheres, Ce; blue spheres, N; magenta spheres, O; purple spheres, protonated O (i.e., OH^−^); gray spheres, C. All hydrogen atoms are omitted for clarity.

The molecular structures of the clusters were determined by single crystal X-ray diffraction (SCXRD) analysis. No counter-ion was observed in the lattice of the clusters, indicating that they were electrically neutral (Tables S1 and S2). The oxidation states of Ce and assignments of O species (O^2−^ versus OH^−^) were confirmed by bond valence sum calculations (Tables S3 and S4). Detailed analysis revealed that **1** crystallized in a triclinic system with a space group of P$\bar{1}$, with only one cluster per unit cell (Figure S2). Cluster **1** consists of a {Ce_16_O_16_(OH)_8_} core with an obvious fluorite structure of bulk CeO_2_, alternating Ce atoms and O atoms, and looking like the fusion of four Ce_6_O_8_ pseudo-dodecahedra (Figure 1a,b). Structurally, like the structure of previously reported [Ce_16_O_17_(OH)_6_(O_2_CPh)_24_(HO_2_CPh)_4_], the four pseudo-dodecahedra in the core of cluster **1** are superimposed on two adjacent clusters by sharing the Ce_2_O_2_ faces, giving rise to fascinating isolated structures with quasi-S_2_ symmetry (Figure 1a). The periphery of the {Ce_16_O_16_(OH)_8_} nucleus in novel cluster **1** is covered with 20 benzoic acid, 8 pyridine, and 2 nitrate ligands, which serve as protecting ligands to stabilize the fascinating isolated structure. The total structure of nanocluster **1** from different directions is shown in Figure 1c,d.

Further dissection and analysis of the structure revealed that the length of Ce-O bonds in the core in cluster **1** exhibits a relatively wide range from 2.102 to 2.680 Å, with an average value of 2.322 Å (for details, see Table S5). It is slightly longer than that in previously reported [Ce_16_O_17_(OH)_6_(O_2_CPh)_24_(HO_2_CPh)_4_], which may be due to the elongation of bond lengths caused by the spatial arrangement of the heterozygous ligands. In addition, the average bond length of Ce-N bonds in **1** is 2.778 Å, and the details are shown in Table 2. Obviously, two Ce ions are not involved in coordination with organic ligands, despite the large variety and number of ligands. The Ce ions whose surfaces are covered by ligands have multiple coordination modes, which are closely related to the involvement of multiple organic ligands. Two of them are 10-coordinated, another six are 8-coordinated, four are in relatively rare 7-coordinations, and last four are 9-coordinated. The ligands consist of 20 monodentate PhCO_2_H, 8 pyridine, and 2 nitrate ligands. A total of 20 of these PhCOO^−^ groups are in the common η^1^: η^1^: μ_2_ chelating and bridging modes. The remaining ligations are provided by two monodentate ligands of nitrate and eight terminal pyridine ligands.

**Figure 2:**
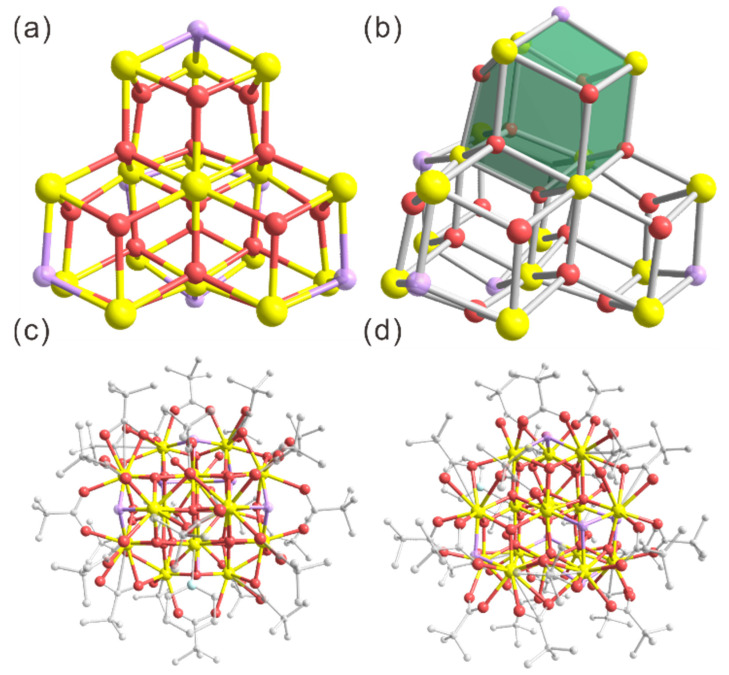
Structural anatomy of **2**: (**a**) {Ce_16_O_17_(OH)_6_} core and (**b**) secondary building units (green pseudo dodecahedra) of **2**; (**c**,**d**) total structure of **2** in the top (**c**) and side view (**d**). Color codes for atoms: yellow spheres, Ce; magenta spheres, O; purple spheres, protonated O (i.e., OH^−^); gray spheres, C. All hydrogen atoms are omitted for clarity.

Cluster **2** crystallized in the *P*2_1_/c space group, with four clusters per unit cell (Figure S3). Cluster **2** consists of a {Ce_16_O_17_(OH)_6_} core (Figure 2a), which can be anatomized as the growth of four Ce_6_O_8_ units via sharing Ce_2_O_2_ faces (Figure 2b). The Ce_6_O_8_ unit is reminiscent of the structure of [Ce_6_O_4_(OH)_4_(dmb)_12_(H_2_O)_4_] [57]. Interestingly, the four Ce_6_O_8_ units share one oxygen atom, which is four-coordinated to the four Ce atoms in the shared Ce_2_O_2_ faces. Moreover, the four-coordinated oxygen atom is in the center of {Ce_16_O_17_(OH)_6_} and the four Ce atoms surrounding it present a fascinating isolated structure with quasi-C_3V_ symmetry (Figure S4). Compared to cluster **1**, the Ce-O bond lengths in the core of **2** are in a somewhat narrower scope, with values between 2.105 and 2.565 Å (see Table S6 for detailed values). The average bond length gives the value of 2.307 Å, which is shorter than that of **1**. The average Ce-N bond length in **2** is 2.672 Å, also shorter than that of **1**. Of note, the {Ce_16_O_17_(OH)_6_} core of cluster **2** is passivated by pivalic acid and a pyridine ligand, which is different from **1**. Moreover, in sharp contrast to **1**, all Ce ions of **2** are exposed on the surface to participate in pivalic acid coordination. The surface Ce ions are ligated by 24 pivalic acids and 1 pyridine. A total of 24 of these C(CH_3_)_3_CO_2_^−^ groups are in the common η^1^: η^1^: μ_2_ bridging mode. In addition, nine Ce ions are in eight-coordination with twisted square antiprismatic geometry, and the remaining seven ones are in nine-coordination with twisted monocapped square antiprismatic geometry (Figure S5) [64,65]. It is noteworthy that the range of the O-Ce-O angle in both the eight-coordination and nine-coordination structures is identical, measuring from 23.2 to 179.4 degrees. The total structure of nanocluster **2** from different directions is shown in Figure 2c,d.

**Figure 3:**
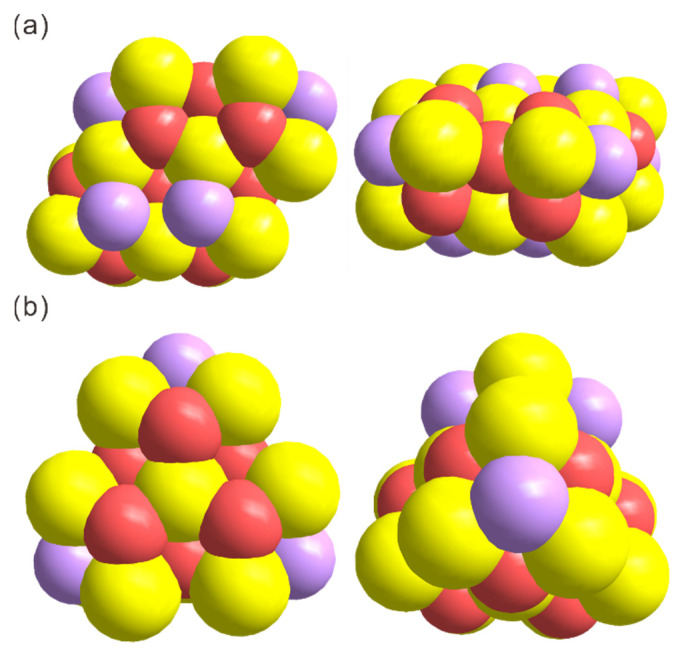
The core structure of Ce_16_ nanoclusters ((**a**) for **1** and (**b**) for **2**) is viewed from different directions. For clarity, all atoms are drawn in a sphere packing mode. Color codes for atoms: yellow spheres, Ce; magenta spheres, O; purple spheres, protonated O (i.e., OH^−^). All other atoms are omitted for clarity.

The kernel structure of the Ce_16_ clusters was further dissected to disclose the effect of encapsulated ligands. To our surprise, both cores of the clusters can be considered assemblies of Ce_6_O_8_ secondary building units (SBUs) and contain 16 Ce atoms. Nevertheless, it turns out their cores show different structures and components (e.g., {Ce_16_O_16_(OH)_8_} for **1** but {Ce_16_O_17_(OH)_6_} for **2**). In cluster **1**, there are still two Ce ions exposed on the surface, while in cluster **2** all Ce ions are capped (Figure 3a,b). Such a difference in the two clusters’ surface structure may lead to a difference in their properties (*vide infra*). What is more, the average bond length of Ce-N and Ce-O in cluster **2** is shorter than that in cluster **1**. It is obvious that the type and amount of surface ligands are not only important for the stabilization of the clusters but also crucial for inducing the formation of different CeO_2_ cluster cores. In other words, the nucleation type of CeO_2_ nanoparticles can be regulated by the modulation of surface ligands, which provides unique structural models for the study of CeO_2_ materials. It further allows the establishment of the composition−structure−property relationships of CeO_2_ and thus facilitates the optimization of their properties for various applications.

**Figure 4:**
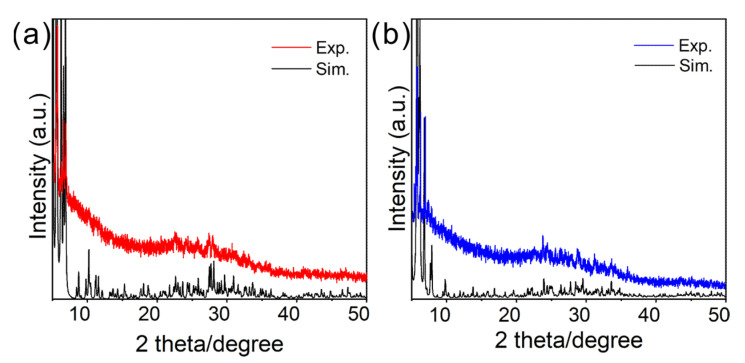
Characterization of the two clusters. (**a**,**b**) Powder X-ray diffraction spectrum of **1** (**a**) and **2** (**b**).

The above-mentioned crystal analysis suggested the neutral state of the clusters. To further verify the proposal, we measured the electrospray ionization mass spectra (ESI-MS) of the clusters (Figures S6 and S7). As shown in Figures S6 and S7, no assignable peak was observed in the ESI-MS spectra of the two clusters in either positive or negative mode. Furthermore, both the powder X-ray diffraction (PXRD) and proton nuclear magnetic resonance (^1^H NMR) studies confirmed their purity (Figures S8 and S9). Displayed in Figure 4a,b is the comparison of experimental and simulated PXRD spectra of **1** and **2**, respectively, in which the high consistency between the measured PXRD spectra and those simulated from the SCXRD verified their purity. The ^1^H NMR analysis of cluster **1** indicates chemical shifts spanning from 7.38 to 8.63 ppm, which can be ascribed to the presence of pyridine (Py) and phenyl (Ph) groups. Furthermore, chemical shifts ranging from 1.11 to 1.27 ppm are identified as corresponding to methyl groups. In contrast, the ^1^H NMR spectrum of cluster **2** exhibits chemical shifts between 7.15 and 8.63 ppm, which are distinctly associated with the Py groups.

**Figure 5:**
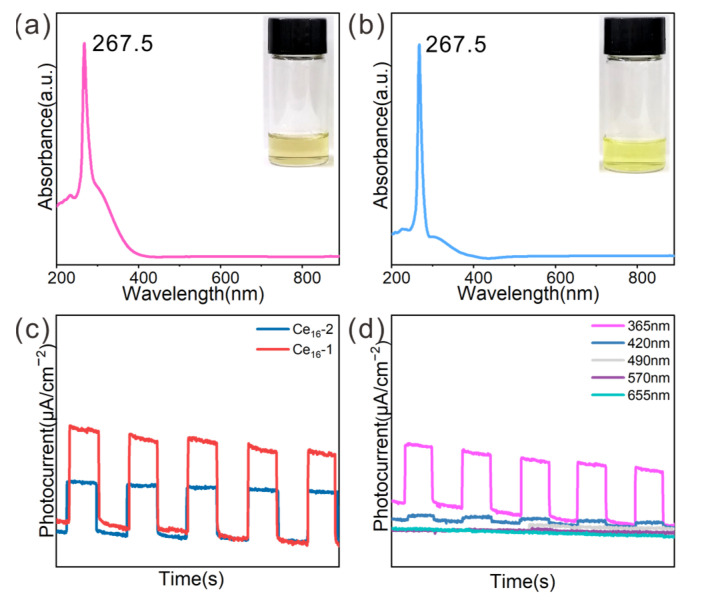
Photoelectric properties of the cluster **1** and **2**. (**a**,**b**) UV–vis spectra of **1** (**a**) and **2** (**b**). Inset is a photograph of the solution; (**c**) the photoelectric response curve of **1** and **2** at a voltage of 1.6 V under light irradiation at 365 nm; (**d**) photoelectric response curve of **1** at a voltage of 1.6 V under irradiation with different wavelengths.

Both clusters **1** and **2** were further analyzed through energy-dispersive X-ray (EDX) mapping. The data showed that Ce, O, C, and N elements of the clusters were evenly distributed on the crystals (Figures S10 and S11). To further explore the optical absorption behavior of **1** and **2**, ultraviolet–visible (UV–vis) spectroscopy was performed at room temperature. As shown in Figure 5a,b, clusters **1** and **2** exhibited unique electronic structures and showed similar optical absorption peaks at around 267.5 nm in N,N-dimethylformamide solution. The yellow color of the solution of the clusters corresponded to the similar color of both crystals. Nevertheless, the optical absorption of the clusters in the range of 300 nm to 400 nm appeared somewhat different, in which the absorption intensity of **1** was stronger than **2**. This suggested that the peripheral ligands might moderately influence the electronic structure of the clusters. For both clusters, moderate stability was observed within 3 h in the solution form (Figure S12).

As discussed above, both **1** and **2** had absorption in the range of 300 nm to 400 nm, and it was assumed that they had a photoelectric response in this wavelength range. The photoelectric response properties of the two clusters were then compared. The cluster samples were dispersed ultrasonically in ethanol and Nafion and then distributed evenly on ITO conductive glass by drip coating. After natural drying, a photoelectric response experiment was carried out.

Photoelectric conversion experiments were carried out with different voltages (0.8 V, 1.0 V, 1.2 V, 1.4 V, 1.6 V, 1.8 V) under light irradiation of 365 nm (Figure S13). The results indicated that the photoelectric conversion efficiencies (PCEs) of **1** and **2** at different voltages under the same light irradiation shared the same trend. Namely, the PCE increased with increasing voltage within a certain range. Both **1** and **2** had the maximum PCE at 1.6 V under light irradiation of 365 nm, indicating that 1.6 V was the optimal voltage. Interestingly, a significant difference in PCE between **1** and **2** was observed under the same measurement conditions (365 nm, 1.6 V). Cluster **1** displayed a PCE 1.8 times that of **2** (Figure 5c). It matched well with the higher light absorption of **1** than that of **2** in the range of 300 nm to 400 nm. The difference in PCE of the two clusters may be caused by the different cluster core structures formed by different ligands. Upon the optimization of voltage (1.6 V), the influence of wavelengths of irradiation light on photocurrent conversion was also investigated. Photoelectric conversion experiments were then performed at 1.6 V under irradiation with different wavelengths (365, 420, 490, 570, and 655 nm). The results showed that there was a maximum PCE at 365 nm. Under irradiation using longer-wavelength lights, a weaker signal was observed. The photocurrent almost disappeared when the wavelength was larger than 490 nm, corresponding to the UV–vis spectra (Figure 5d and Figure S14).

## 3. Experimental

Materials and Reagents: Pyridine (C_5_H_5_N, 99.5%) was purchased from Tianjin Fengchuan Chemical Reagent Co., Ltd. (Tianjin, China). Ammonium ceric nitrate (CeH_8_N_8_O_18_, 99%) was purchased from Shanghai Acmec Biochemical Co., Ltd. (Shanghai, China). Benzoic acid (C_7_H_6_O_2_, 99.45%) was purchased from Bidepharm (Shanghai, China). Pivalic acid (C_5_H_10_O_2_, 99%) was purchased from J&K Scientific Ltd. (Beijing, China). N,N-Dimethylformamide (DMF, C_3_H_7_NO, 99.5%), ethanol (C_2_H_6_O, A.R.), methanol (CH_3_OH, 99.5%), and acetonitrile (C_2_H_3_N, A.R.) were purchased from Sinopharm Chemical Reagent Co., Ltd. (Shanghai, China). All other reagents were used as received without further purification.

### Methods

Synthesis of **1**: Ammonium ceric nitrate (550 mg, 1.003 mmol) and benzoic acid (225 mg, 1.842 mmol) were dissolved in 10 mL of pyridine, followed by the addition of 8.1 µL of methanol. After stirring for 30 min, the suspension was centrifuged, and the unreacted residue was discarded. The above solution was transferred into a 25 mL colorimetric tube and layered with 20 mL of acetonitrile. After being kept undisturbed in a dark environment for 2 weeks, yellow crystals were obtained. Yield: 4.4% based on Ce with some unknown powder precipitated. Compound **2** was synthesized by a similar method to **1**, except for the absence of methanol and the use of pivalic acid in the synthesis. Yield: 4.7% based on Ce with some unknown powder precipitated.

Preparation of the working electrode for photocurrent response measurements: The clusters (6 mg) were dispersed ultrasonically in ethanol (970 µL) and Nafion (30 µL) to generate a homogeneous slurry. Subsequently, 40 µL of the slurry was transferred and coated on ITO glass plates (1 cm × 2 cm) and then dried at room temperature.

Photoelectrochemical Experiments: A single-compartment cell with a three-electrode configuration was employed in this study. The cluster-coated ITO glass was used as a working electrode, a platinum wire as a counter electrode, and a saturated Ag/AgCl electrode (with saturated KCl solution) as a reference electrode. A 0.5 M Na_2_SO_4_ aqueous solution was used as the electrolyte. Photocurrent response performance was evaluated under irradiation with different monochromatic LED light sources of various wavelengths (50 W). The light source was 10 cm away from the quartz cell.

General Characterizations: UV–vis spectra were collected using a JascoV-650 spectrophotometer at room temperature (Japan Spectroscopy Co., Ltd. JASCO Corporation, Tokyo, Japan). Electrospray ionization mass spectra (ESI-MS) were recorded using an Agilent 6224 time-of-flight mass spectrometer (Agilent Technology Co., Ltd., Santa Clara, CA, USA). ^1^H NMR spectra were collected at room temperature on a Bruker AV-600 spectrometer with TMS and solvent residual signal as an internal reference. The NMR data were processed in MestReNova software (11.0). The EDS elemental mapping studies were performed on a TECNAI F30 transmission electron microscope operating at 300 kV (Field Electron and Ion Company, Hillsboro, OR, USA).

Crystal Structure Determinations: For the X-ray single-crystal analysis, the diffraction data of the single crystals of **1** and **2** were collected on a Rigaku Oxford Diffraction system X-ray single-crystal diffractometer (Rigaku Corporation, Tokyo, Japan) using Cu Kα (λ = 1.54184 Å) at 100 K. The data were processed using CrysAlis^Pro^ (Rigaku Corporation, Tokyo, Japan). The structure was solved and refined using full-matrix least-squares based on F2 using ShelXT (http://shelx.uni-goettingen.de/) [66] and ShelXL (http://shelx.uni-goettingen.de/) [67] in Olex2 (1.3) [68]. The thermal ellipsoids of the ORTEP diagram were obtained at 50% probability. Detailed crystal data and structural refinements for the compound are given in Tables S1 and S2. The CCDC numbers 2337609 (**1**) and 2337611 (**2**) contain the supplementary crystallographic data for this study.

## 4. Conclusions

In summary, the ligand strategy for regulating CeO_2_ nanoparticles in terms of their composition, structure, and properties is reported. By employing two kinds of carboxylic acids (benzoic acid and pivalic acid) in the synthesis, two atomically precise Ce_16_ nanoparticles ([Ce_16_O_16_(OH)_8_(O_2_CPh)_20_(py)_8_(NO_3_)_2_] and [Ce_16_O_17_(OH)_6_(O_2_C^t^Bu)_24_(py)]) are gained. The two nanoparticles (also called nanoclusters) exhibit different compositions, shapes, core structures, Ce^3+^/Ce^4+^ ratios, and photoelectric properties. The ligand strategy demonstrated here may be applicable to more CeO_2_ and even other metal oxide materials. More effort, including employing the two Ce_16_ clusters in the field of CMP, studying the fundamental issue of structure–property relationships of CeO_2_ materials, and exploring more atomically precise rare earth oxides, are ongoing in our laboratories.

## Figures and Tables

**Table 1:** Selective synthetic parameters of Ce_16_ clusters.

Prototypes	Miscible-Phase Solution	
Compounds	**1**	**2**
Precursors	(NH_4_)_2_[Ce(NO_3_)_6_] + PhCO_2_H	(NH_4_)_2_[Ce(NO_3_)_6_] + C(CH_3_)_3_COOH
Solvents	Py + CH_3_OH	Py
Crystallization	Diffusion	Diffusion

**Table 2:** Average bond lengths of some Ce-O compounds.

Bonds		Ce-O/Å	Ce-N/Å
Compounds	**1**	2.322	2.778
	**2**	2.307	2.672

## Data Availability

All data are contained within the article or Supplementary Materials.

## Supplementary Materials

The following supporting information can be downloaded at https://www.mdpi.com/article/10.3390/molecules30040846/s1: Figure S1: Photographs of single crystals of Ce_16_ clusters **1** and **2**; Figures S2 and S3: The packing structure of **1** and **2**; Figure S4: Ce_4_O tetrahedra in the center of the cluster; Figure S5: The geometry of the 8- and 9-coordination; Figures S6 and S7: ESI-MS of **1** and **2**; Figures S8 and S9: ^1^H NMR of **1** and **2**; Figures S10 and S11: EDS mapping of **1** and **2**; Figure S12: Time-dependent UV–vis spectra of **1** and **2**; Figure S13: The photoelectric response of **1** and **2** at different voltages under the wavelength of 365 nm; Figure S14: The photoelectric response of **2** under different wavelengths at 1.6 V; Tables S1 and S2: Crystal data and structure refinement of **1** and **2**; Table S3: Bond valence sum calculations for cerium centers in **1** and **2**; Table S4: Bond valence sum calculations for *μ*_3_-O and *μ*_4_-O atoms in **1** and **2**; Tables S5 and S6: Selected bond lengths (Å) for compound **2**.
