# Supplementary material for: Ligand Strategies for Regulating Atomically Precise CeO2 Nanoparticles: From Structure to Property
Source: Molecules. 2025 Feb 12;30(4):846. doi: 10.3390/molecules30040846 (PMC11858138; doi:10.3390/molecules30040846)
Supplement: Supplementary file 1 [file molecules-30-00846-s001.zip › molecules-3395172-supplementary.pdf]

Supplementary Materials

Ligand Strategies for Regulating Atomically Precise CeO_2 Nanoparticles: from Structure to Property

Peiling Du,[1,†] Simin Li,[1,†] Qinghua Xu,[1] Ayisha He,[1] Wei Yuan,[2] Xin-Ping Qu,[3] Baimei Tan,[4] Xinhuan Niu,[4] Hui Shen,[1,*] and Fan Zhang[1,*]

1 College of Energy Materials and Chemistry, Inner Mongolia University, Hohhot 010021, China

2 Shanghai Frontiers Science Research Base of Intelligent Optoelectronics and Perception, Institute of Optoelectronics, Fudan University, 2005 Songhu Road, Shanghai, 200438, China

3 School of Microelectronics, Fudan University, Shanghai, 200433, China

4 School of Electronics and Information Engineering, Hebei University of Technology, Tianjin 300130, China

* Correspondence: shen@imu.edu.cn; Zhang_fan@fudan.edu.cn

† These authors contributed equally to this work.

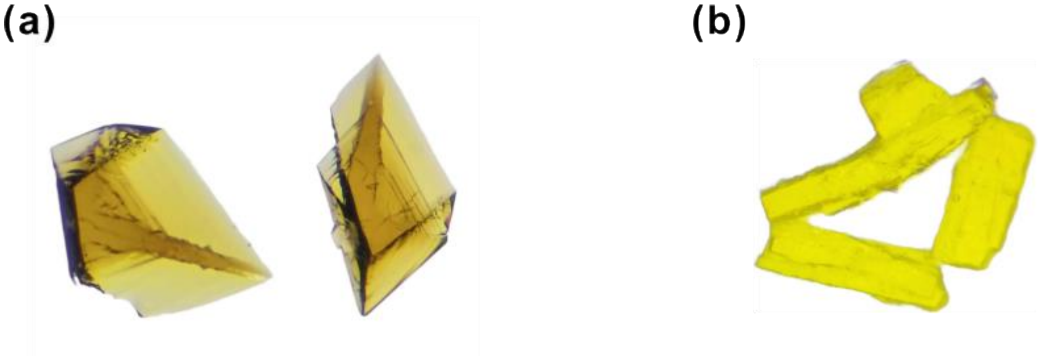

Figure S1. Photographs of single crystals of Ce_{16} clusters of **1** (a) and **2** (b).

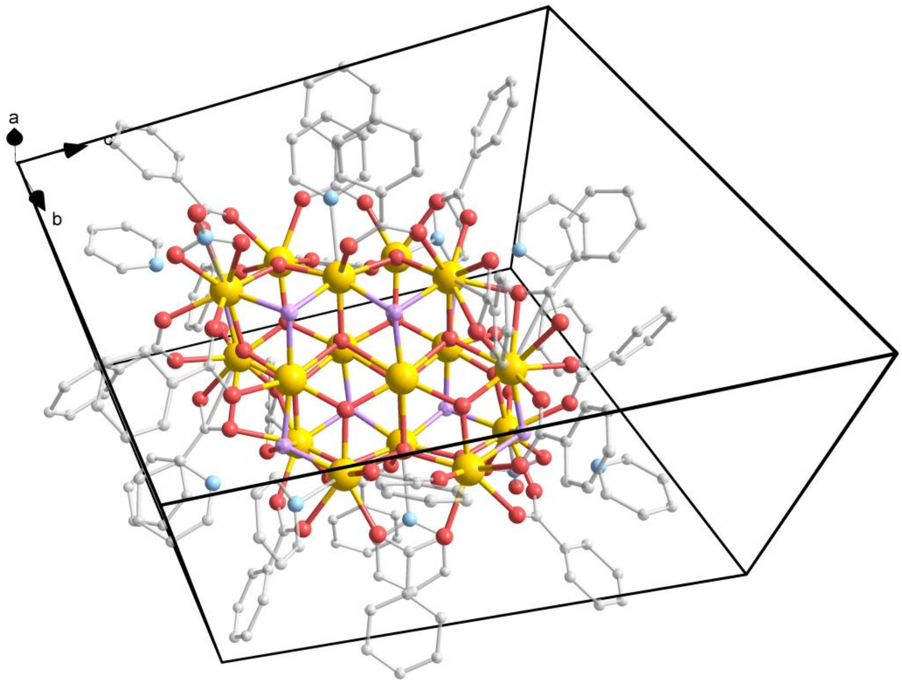

Figure S2. The packing structure of **1** in their single crystals. Colour codes for atoms: yellow spheres, Ce; blue spheres, N; magenta spheres, O; purple spheres, protonated O (i.e., OH^-); grey spheres, C. All hydrogen atoms are omitted for clarity.

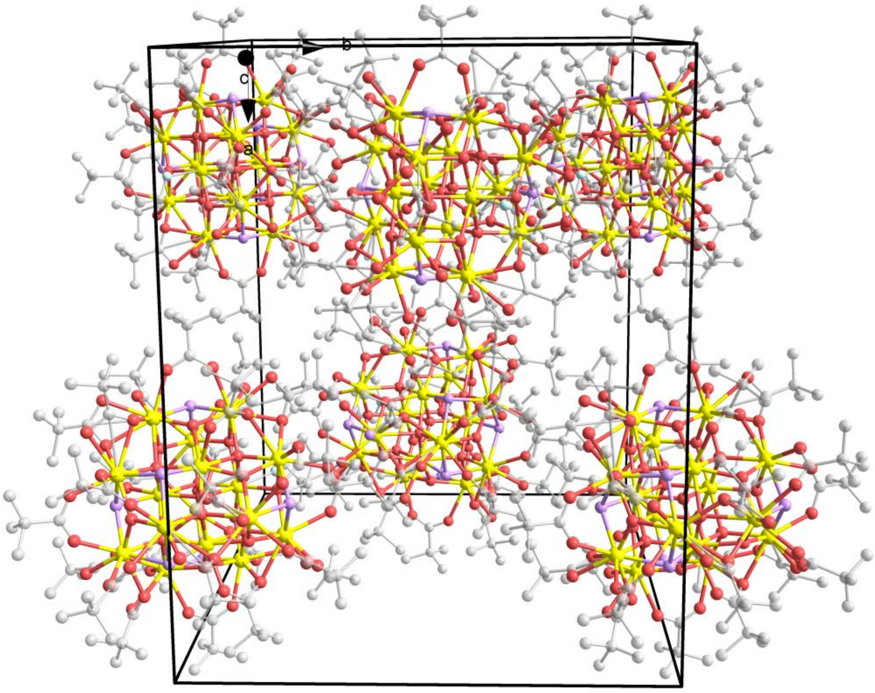

Figure S3. The packing structure of **2** in their single crystals. Colour codes for atoms: yellow spheres, Ce; blue spheres, N; magenta spheres, O; purple spheres, protonated O (i.e., OH^-); grey spheres, C. All hydrogen atoms are omitted for clarity.

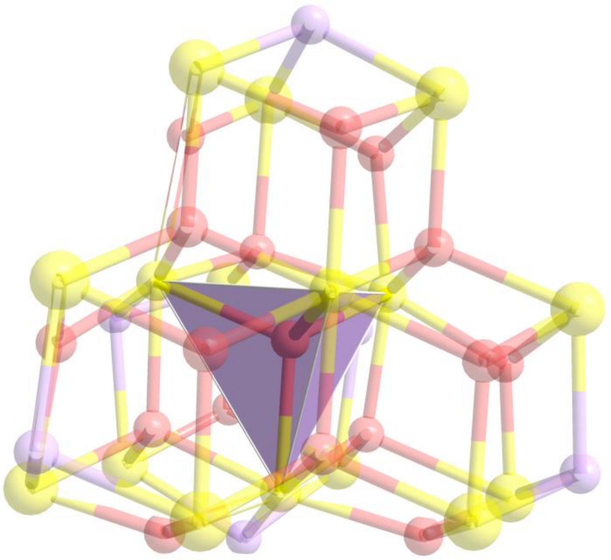

Figure S4. Ce_4O tetrahedra in the centre of the cluster. Colour codes for atoms: yellow spheres, Ce; magenta spheres, O; purple spheres, protonated O (i.e., OH^-).

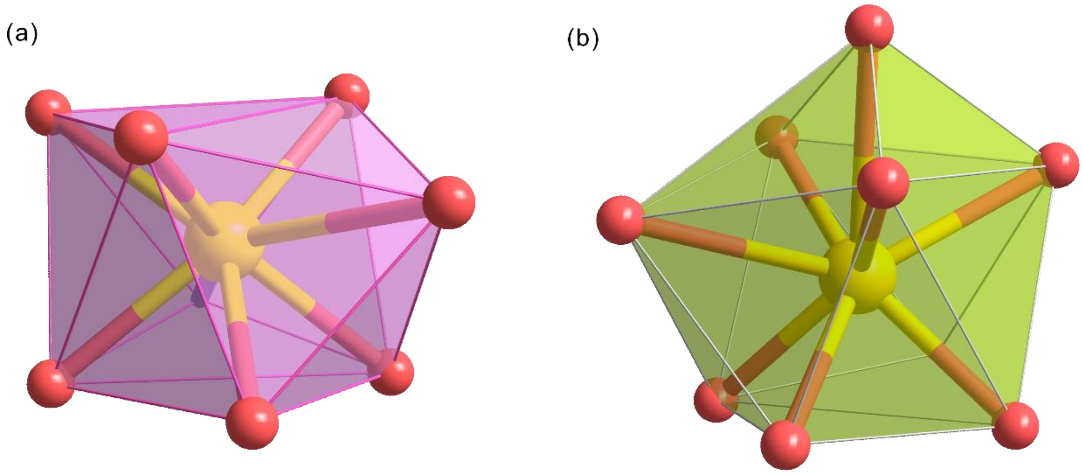

Figure S5. (a) The twisted square antiprism geometry of the 8-coordination Ce; (b) The twisted monocapped square antiprism of the 9-coordination Ce. Colour codes for atoms: yellow spheres, Ce; magenta spheres, O; purple spheres, protonated O (i.e., OH^-).

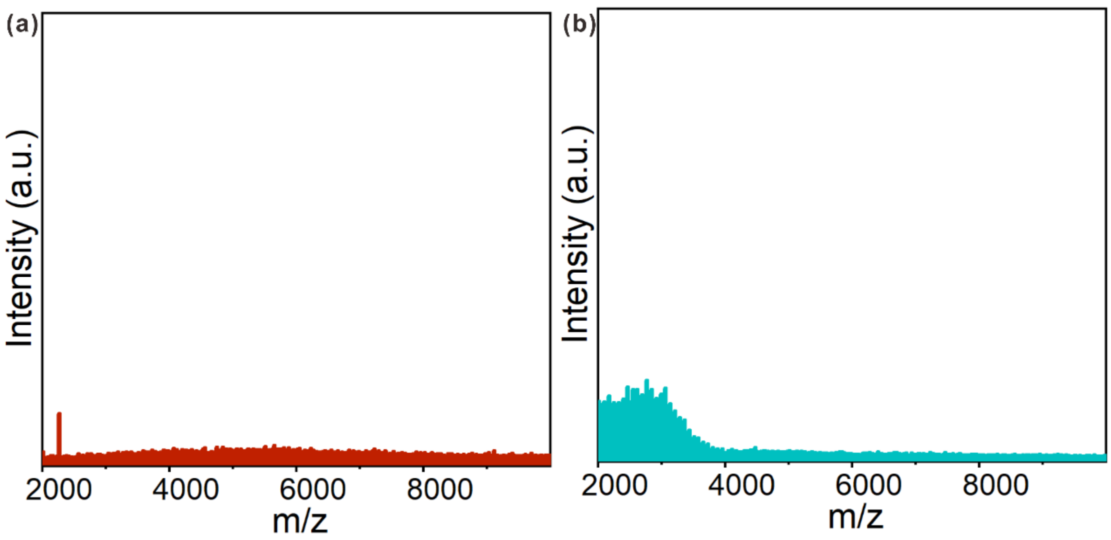

Figure S6. (a) ESI-MS of **1** in the negative mode; (b) ESI-MS of **1** in the positive mode.

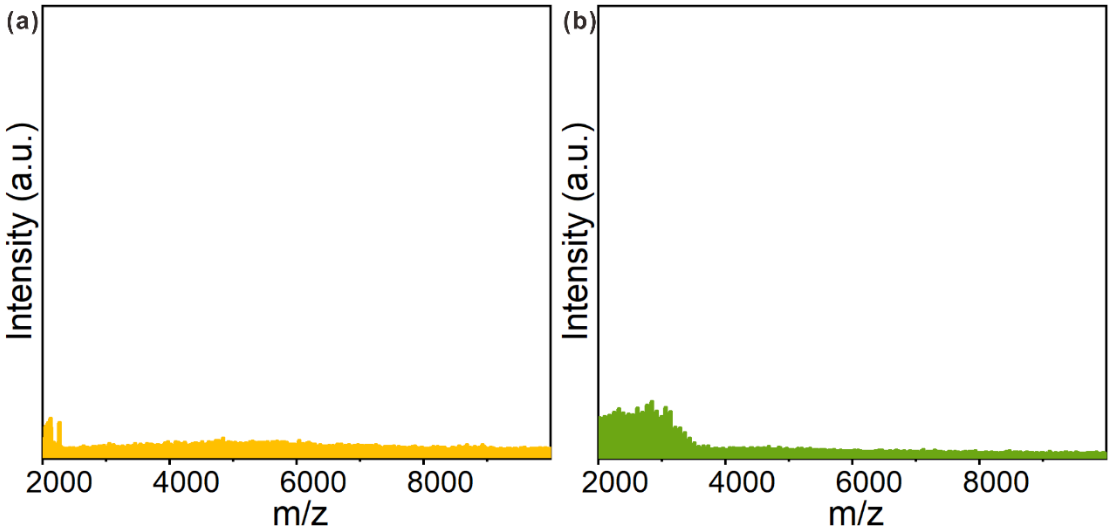

Figure S7. (a) ESI-MS of **2** in the negative mode; (b) ESI-MS of **2** in the positive mode.

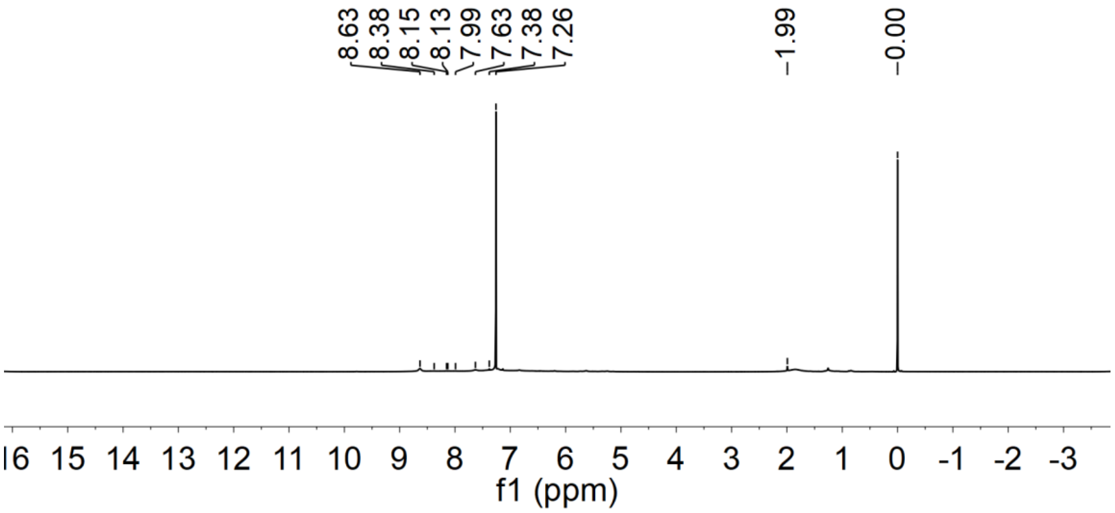

Figure S8. ^{1}H NMR of **1** in $CDCl_3$.

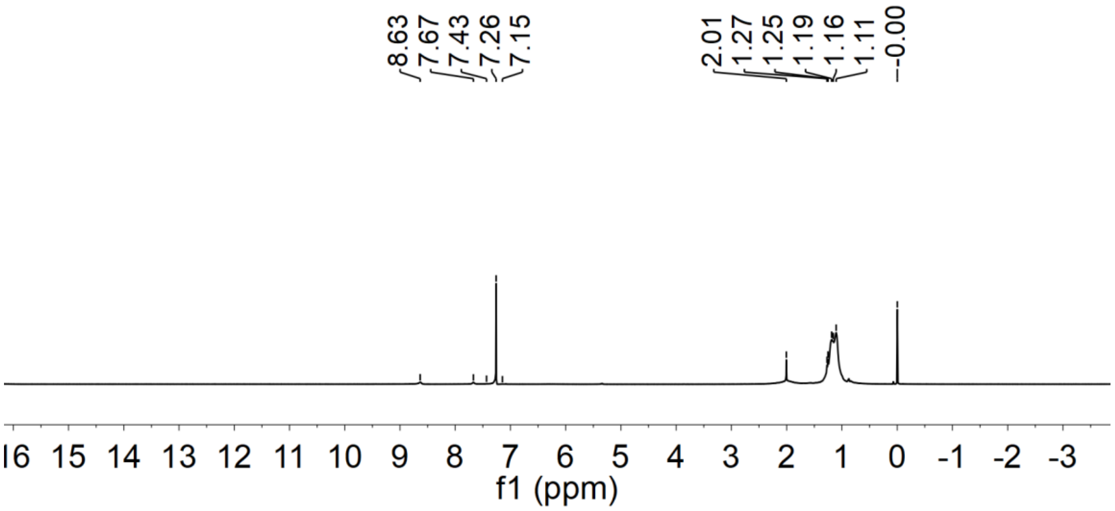

Figure S9. ^{1}H NMR of **2** in $CDCl_3$.

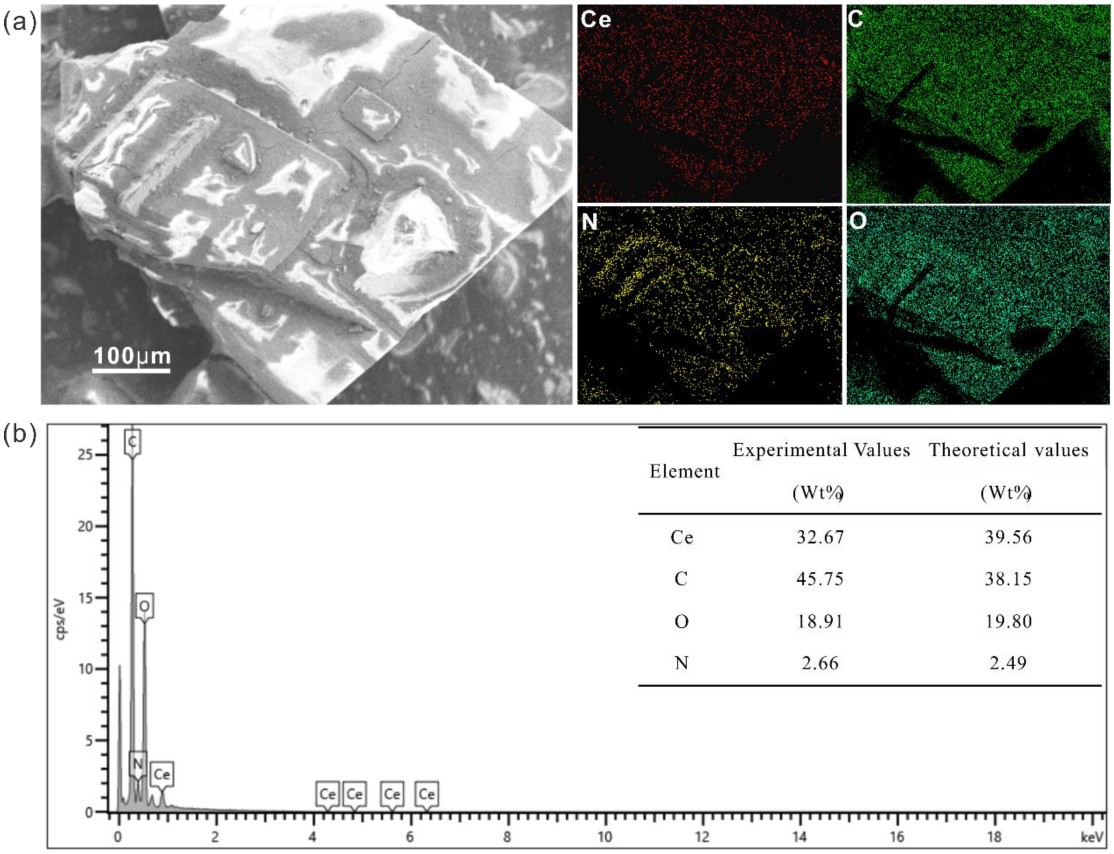

Element	Experimental Values (Wt%)	Theoretical values (Wt%)
Ce	32.67	39.56
C	45.75	38.15
O	18.91	19.80
N	2.66	2.49

Figure S10. (a) EDS mapping of **1**; (b) EDS spectrum of **1**, inset: the weight percentage value found for the elements.

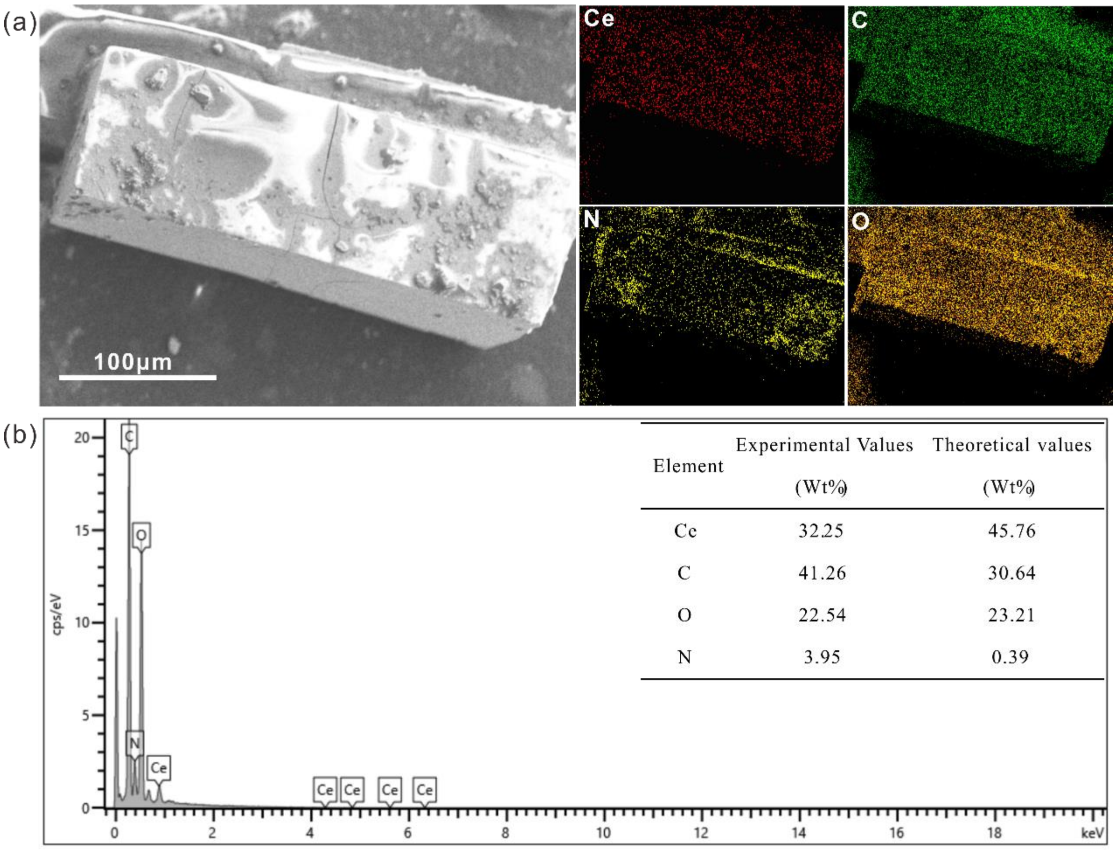

Element	Experimental Values (Wt%)	Theoretical values (Wt%)
Ce	32.25	45.76
C	41.26	30.64
O	22.54	23.21
N	3.95	0.39

Figure S11. (a) EDS mapping of **2**; (b) EDS spectrum of **2**, inset: the weight percentage value found for the elements.

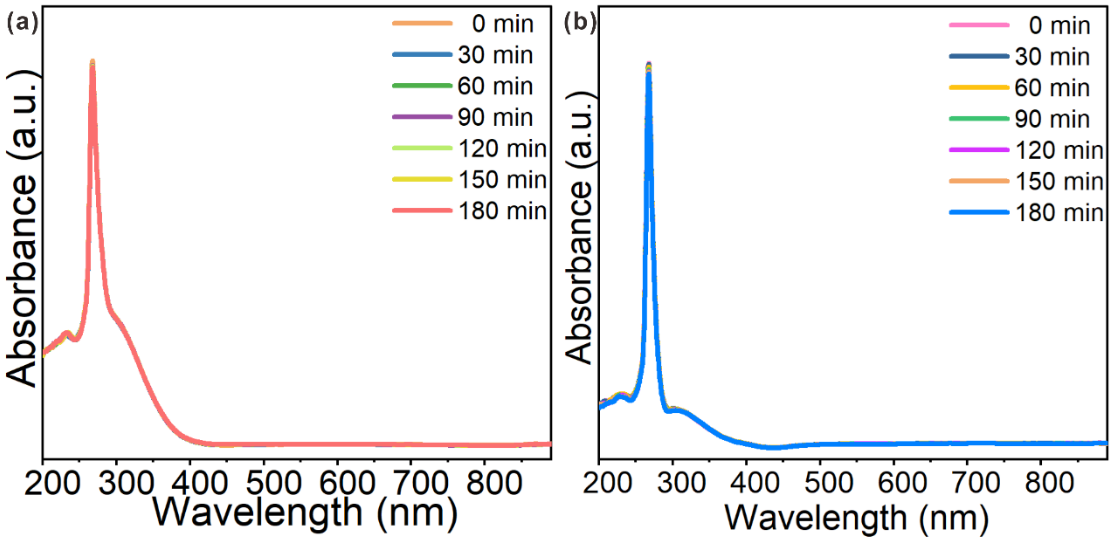

Figure S12. Time-dependent UV-vis spectra of **1** (a) and **2** (b).

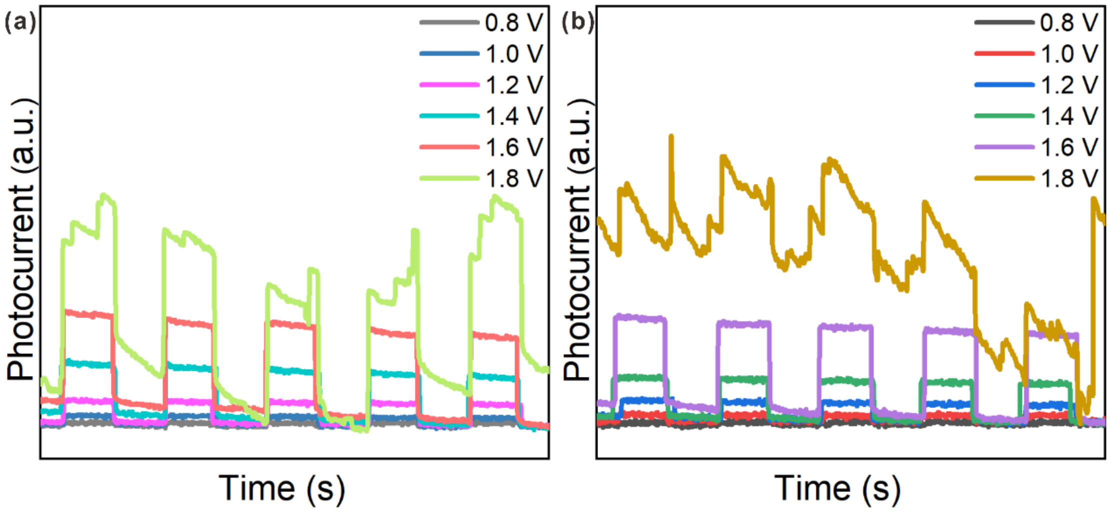

Figure S13. The photoelectric response of **1** (a) and **2** (b) at different voltages under the wavelength of 365 nm.

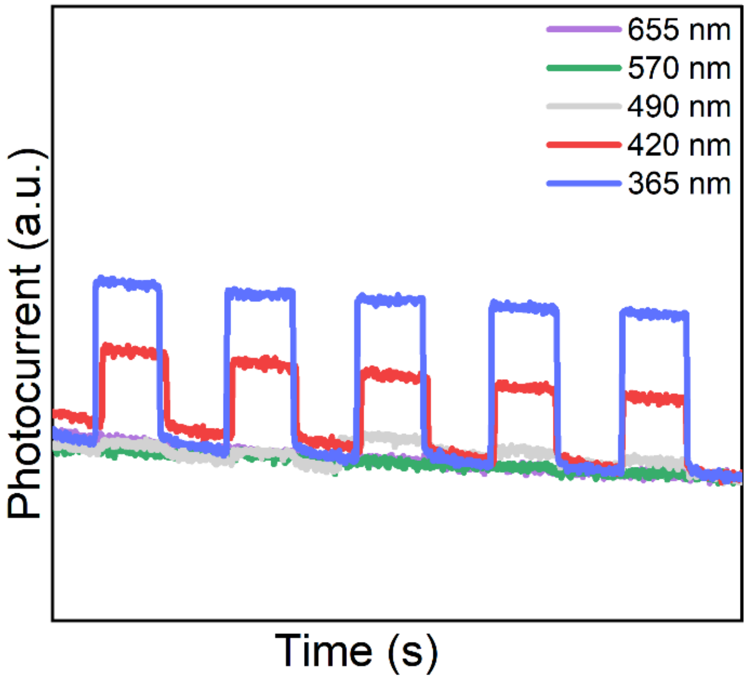

Figure S14. The photoelectric response of **2** under different wavelengths at 1.6 V.

Table S1. Crystal data and structure refinement of **1**.

Compound	$[Ce_{16}O_{16}(OH)_8(O_2CPh)_{20}(py)_8(NO_3)_2]$
CCDC No.	2337609
Formula	$C_{180}H_{148}Ce_{16}N_{10}O_{70}$
Formula weight	5813.00
Temperature/K	100.01(10)
Crystal system	triclinic
Space group	P-1
a (Å)	17.4756(17)
b (Å)	18.301(2)
c (Å)	19.9291(14)
α (°)	63.967(9)
β (°)	68.543(8)
γ (°)	68.503(10)
V ($Å^3$)	5166.0(10)
Z	1
D_c / ($g·cm^{-3}$)	1.869
Radiation	Cu Kα (λ = 1.54184)
Theta (°) range	6.586 to 79.94
Index ranges	$-14 \le h \le 14$, $-15 \le k \le 14$, $-16 \le l \le 10$
Refls. Total	16102
Restraints	1625
Parameters	1111
R_{int}	0.1040
R_1/wR_2 [I>2σ(I)]	0.0652 /0.1507
R_1/wR_2 (all data)	0.1101 / 0.1917
Goodness-of-fit on F^2	0.999

Table S2. Crystal data and structure refinement of **2**.

Compound	$[Ce_{16}O_{17}(OH)_6(O_2CtBu)_{24}(py)]$
CCDC No.	2337611
Formula	$C_{125}H_{227}Ce_{16}NO_{71}$
Formula weight	5121.98
Temperature/K	100.00(10)
Crystal system	monoclinic
Space group	$P2_1/c$
a (Å)	28.6206(3)
b (Å)	23.0378(3)
c (Å)	29.2285(4)
α (˚)	90
β (˚)	90.0630(10)
γ (˚)	90
V ($Å^3$)	19272.0(4)
Z	4
D_c / ($g·cm^{-3}$)	1.765
Radiation	Cu Kα (λ = 1.54184)
Theta (˚) range	4.884 to 108.484
Index ranges	$-30 \leq h \leq 27$, $-24 \leq k \leq 22$, $-28 \leq l \leq 30$
Refls. Total	153739
Restraints	2384
Parameters	1978
R_{int}	0.1451
R_1/wR_2 [I>2σ(I)]	0.1229 / 0.3251
R_1/wR_2 (all data)	0.1460 / 0.3469
Goodness-of-fit on F^2	1.034

Table S3. Bond valence sum calculations for cerium centres in **1** and **2.**

Clusters	Atoms	Ce^{III}	|d|	Ce^{IV}	|d|
1	Ce01	4.354	1.354	3.824	**0.176**
	Ce02	3.777	0.777	3.317	0.683
	Ce03	4.540	1.540	3.988	**0.012**
	Ce04	4.404	1.404	5.282	1.282
	Ce05	4.533	1.533	3.982	**0.018**
	Ce06	4.538	1.538	5.516	1.516
	Ce07	4.211	1.211	5.539	1.539
	Ce08	4.259	1.259	3.741	**0.259**
2	Ce01	4.312	1.312	5.248	1.248
	Ce02	4.632	1.632	5.525	1.525
	Ce03	4.448	1.448	5.249	1.249
	Ce04	4.503	1.503	5.248	1.248
	Ce05	4.471	1.471	5.287	1.287
	Ce06	4.473	1.473	3.928	**0.072**
	Ce07	4.514	1.514	5.282	1.282
	Ce08	4.213	1.213	3.700	**0.300**
	Ce09	4.260	1.260	3.742	**0.258**
	Ce0A	3.922	0.922	3.445	0.555
	Ce0B	4.434	1.434	3.895	**0.105**
	Ce0C	4.407	1.407	3.871	**0.129**
	Ce0D	4.298	1.298	3.775	**0.225**
	Ce0E	4.298	1.298	3.775	**0.225**
	Ce0F	4.316	1.316	3.791	**0.209**

Note: The bold part can be accurately classified as CeIV, and the remaining atoms may appear as mixed valence states

Table S4. Bond valence sum calculations for μ_3-O and μ_4-O atoms in **1** and **2**.

Clusters	Atoms	BVS	Assignment
1	O00T	1.081	OH^-
	O00E	1.250	OH^-
	O015	1.271	OH^-
	O012	1.072	OH^-
	O00H	2.056	O^{2-}
	O009	1.871	O^{2-}
	O00N	1.985	O^{2-}
	O00J	2.085	O^{2-}
	O00B	2.054	O^{2-}
	O00F	1.884	O^{2-}
	O00D	2.020	O^{2-}
	O00L	2.093	O^{2-}
2	O00P	1.050	OH^-
	O00X	1.035	OH^-
	O011	0.970	OH^-
	O01R	1.361	OH^-
	O014	1.371	OH^-
	O01N	1.375	OH^-
	O00I	2.246	O^{2-}
	O00K	2.230	O^{2-}
	O010	2.044	O^{2-}
	O00U	2.107	O^{2-}
	O00L	2.153	O^{2-}
	O00J	1.861	O^{2-}
	O00H	1.982	O^{2-}
	O00N	1.970	O^{2-}

O00V	2.026	O^{2-}
O00M	1.971	O^{2-}
O00S	1.999	O^{2-}
O00O	2.009	O^{2-}
O00T	2.061	O^{2-}
O019	2.051	O^{2-}
O01L	2.039	O^{2-}
O00Q	1.909	O^{2-}
O00Y	1.859	O^{2-}

Table S5. Selected bond lengths (Å) for compound **1**.

Parameter	value	Parameter	value
**Ce01-Ce02**	3.794(2)	Ce04-O00X	2.424(18)
**Ce01-Ce02[1]**	3.789(3)	Ce04-O019	2.28(2)
**Ce01-Ce03[1]**	3.758(3)	Ce05-O00E	2.38(2)
**Ce01-Ce05[1]**	3.738(3)	Ce05-O00G	2.37(2)
**Ce01-Ce06**	3.718(3)	Ce05-O00L[1]	2.26(2)
**Ce01-O00D**	2.31(2)	Ce05-O00M	2.430(16)
**Ce01-O00F[1]**	2.376(16)	Ce05-O00N	2.166(17)
**Ce01-O00J**	2.163(17)	Ce05-O00S	2.50(2)
**Ce01-O00L**	2.102(18)	Ce05-O00Y	2.32(2)
**Ce01-O00T**	2.352(18)	Ce05-O012[1]	2.506(18)
**Ce01-O00W[1]**	2.48(2)	Ce05-O017	2.62(2)
**Ce01-O012**	2.420(19)	Ce05-N01A	2.99(3)
**Ce02-Ce03**	3.733(2)	Ce06-Ce07	3.693(3)
**Ce02-Ce07**	3.745(3)	Ce06-Ce08	3.796(3)
**Ce02-Ce08[1]**	3.778(2)	Ce06-O009	2.275(15)
**Ce02-O009**	2.569(19)	Ce06-O00B	2.15(2)
**Ce02-O00D[1]**	2.342(16)	Ce06-O00J	2.230(19)
**Ce02-O00F**	2.25(2)	Ce06-O00K	2.32(2)
**Ce02-O00F[1]**	2.619(17)	Ce06-O00T	2.378(17)
**Ce02-O00H**	2.261(17)	Ce06-O00Z	2.519(16)
**Ce02-O00J**	2.226(15)	Ce06-O011	2.40(2)
**Ce02-O015**	2.332(19)	Ce06-N01H	2.625(16)
**Ce03-Ce04**	3.720(2)	Ce07-Ce08	3.761(2)
**Ce03-O00D[1]**	2.427(17)	Ce07-O009	2.31(2)
**Ce03-O00E**	2.36(2)	Ce07-O00B	2.266(16)
**Ce03-O00H**	2.168(16)	Ce07-O00F	2.262(19)
**Ce03-O00I[1]**	2.52(2)	Ce07-O00N	2.258(16)

Ce03 -O00L[1]	2.28(2)	Ce07-O012[1]	2.405(18)
**Ce03-O00P[1]**	2.514(17)	Ce07-O014	2.40(2)
**Ce03-O00R[1]**	2.63(2)	Ce07-O015[1]	2.461(18)
**Ce03-O00U**	2.29(2)	Ce07-N00O	2.720(14)
**Ce03-O00V**	2.373(19)	Ce08-O00B	2.217(18)
**Ce03-C01R[1]**	2.94(2)	Ce08-O00C	2.27(2)
**Ce04-Ce05**	3.757(3)	Ce08-O00D	2.23(2)
**Ce04-Ce07**	3.707(2)	Ce08-O00I	2.64(2)
**Ce04-O009**	2.340(16)	Ce08-O00R	2.490(18)
**Ce04-O00A**	2.423(18)	Ce08-O00T	2.68(2)
**Ce04-O00E**	2.423(16)	Ce08-O015[1]	2.354(16)
**Ce04-O00H**	2.21(2)	Ce08-O016	2.49(2)
**Ce04-O00N**	2.25(2)	Ce08-O018	2.45(2)
**Ce04-O00Q**	2.43(2)	Ce08-C03D	2.89(2)

Table S6. Selected bond lengths (Å) for compound **2**.

**Parameter**	**value**	**Parameter**	**value**
**Ce01-Ce02**	3.8071(19)	Ce08-O025	2.35(3)
**Ce01-Ce05**	3.701(2)	Ce08-C03F	3.04(5)
**Ce01-Ce06**	3.7226(19)	Ce08-O04H	2.77(3)
**Ce01-Ce0B**	3.783(2)	Ce08-O051	2.43(3)
**Ce01-O00H**	2.333(13)	Ce09-Ce0D	3.819(2)
**Ce01-O00I**	2.362(16)	Ce09-O00M	2.188(17)
**Ce01-O00M**	2.325(15)	Ce09-O00P	2.470(16)
**Ce01-O00O**	2.355(16)	Ce09-O00X	2.514(17)
**Ce01-O00R**	2.432(18)	Ce09-O01C	2.60(2)
**Ce01-O00S**	2.287(18)	Ce09-O01F	2.63(2)
**Ce01-O00V**	2.282(15)	Ce09-O01L	2.195(19)
**Ce01-O00X**	2.415(17)	Ce09-O01V	2.37(2)
**Ce02-Ce05**	3.719(2)	Ce09-O020	2.36(3)
**Ce02-Ce06**	3.758(2)	Ce09-O02A	2.50(3)
**Ce02-Ce07**	3.750(2)	Ce09-C02T	2.91(4)
**Ce02-Ce0E**	3.753(2)	Ce0A-Ce0G	3.806(2)
Ce02- O00H	2.340(15)	Ce0A-O00N	2.243(15)
**Ce02-O00I**	2.271(15)	Ce0A-O00P	2.472(18)
**Ce02-O00J**	2.289(17)	Ce0A-O011	2.483(19)
**Ce02-O00K**	2.315(13)	Ce0A-O017	2.39(2)
**Ce02-O00L**	2.280(14)	Ce0A-O01I	2.67(3)
**Ce02-O00Q**	2.309(18)	Ce0A-O01L	2.272(18)
**Ce02-O00Y**	2.283(15)	Ce0A-O01O	2.55(2)
**Ce02-N2K**	2.671(17)	Ce0A-O01Y	2.41(2)
**Ce03-Ce04**	3.7930(19)	Ce0A-O035	2.52(3)
**Ce03-Ce07**	3.676(2)	Ce0A-C2A	2.97(3)
**Ce03-Ce0F**	3.727(2)	Ce0B-O00O	2.468(16)

**Ce03-Ce0G**	3.799(2)	Ce0B-O00T	2.173(17)
**Ce03-O00H**	2.341(14)	Ce0B-O00V	2.173(18)
**Ce03-O00K**	2.353(13)	Ce0B-O014	2.374(18)
**Ce03-O00N**	2.296(13)	Ce0B-O01P	2.54(2)
**Ce03-O00O**	2.343(15)	Ce0B-O01Q	2.62(2)
**Ce03-O00T**	2.236(15)	Ce0B-O01W	2.38(3)
**Ce03-O010**	2.303(17)	Ce0B-O04H	2.50(3)
**Ce03-O011**	2.431(18)	Ce0B-O5	2.47(3)
**Ce03-O01T**	2.41(2)	Ce0B-C25	2.94(3)
**Ce04-Ce0C**	3.693(2)	Ce0C-O00L	2.306(15)
**Ce04-Ce0E**	3.684(2)	Ce0C-O00Q	2.208(17)
**Ce04-Ce0G**	3.785(2)	Ce0C-O00W	2.41(2)
**Ce04-O00H**	2.307(13)	Ce0C-O015	2.37(2)
**Ce04-O00L**	2.319(16)	Ce0C-O019	2.226(17)
**Ce04-O00M**	2.342(16)	Ce0C-O01G	2.63(2)
**Ce04-O00N**	2.331(15)	Ce0C-O01N	2.402(16)
**Ce04-O00P**	2.424(17)	Ce0C-O021	2.54(3)
**Ce04-O00U**	2.26(2)	Ce0C-O1	2.58(4)
**Ce04-O019**	2.256(19)	Ce0C-C1Y	2.93(3)
**Ce04-O01Z**	2.45(2)	Ce0D-O00M	2.565(16)
**Ce05-Ce0C**	3.696(2)	Ce0D-O00S	2.142(15)
**Ce05-Ce0D**	3.737(2)	Ce0D-O019	2.157(19)
**Ce05-O00I**	2.287(15)	Ce0D-O01C	2.57(2)
**Ce05-O00Q**	2.206(16)	Ce0D-O01D	2.46(2)
**Ce05-O00S**	2.247(17)	Ce0D-O01F	2.53(2)
**Ce05-O00Z**	2.664(18)	Ce0D-O01H	2.50(3)
**Ce05-O018**	2.31(2)	Ce0D-O01N	2.40(2)
**Ce05-O01B**	2.40(2)	Ce0D-O031	2.54(3)
**Ce05-O01M**	2.45(2)	Ce0D-C04V	3.08(4)

**Ce05-O01N**	2.28(2)	Ce0E-Ce0F	3.722(3)
**Ce06-Ce07**	3.718(2)	Ce0E-Ce0G	3.737(3)
**Ce06-O00I**	2.277(16)	Ce0E-O00J	2.230(18)
**Ce06-O00V**	2.201(17)	Ce0E-O00L	2.291(15)
**Ce06-O00Y**	2.238(16)	Ce0E-O00U	2.26(2)
**Ce06-O00Z**	2.632(18)	Ce0E-O01G	2.667(18)
**Ce06-O012**	2.42(2)	Ce0E-O01R	2.31(2)
**Ce06-O014**	2.40(2)	Ce0E-O021	2.50(3)
**Ce06-O01E**	2.29(2)	Ce0E-O024	2.43(3)
**Ce06-O01M**	2.64(2)	Ce0E-O027	2.32(3)
**Ce06-O02C**	2.55(2)	Ce0F-O00J	2.284(19)
**Ce06-C037**	2.99(4)	Ce0F-O00K	2.270(14)
**Ce07-Ce0B**	3.733(2)	Ce0F-O010	2.242(19)
**Ce07-O00K**	2.268(13)	Ce0F-O013	2.63(2)
**Ce07-O00T**	2.222(17)	Ce0F-O01K	2.64(2)
**Ce07-O00Y**	2.224(16)	Ce0F-O01R	2.386(18)
**Ce07-O013**	2.45(3)	Ce0F-O022	2.45(3)
**Ce07-O014**	2.301(18)	Ce0F-O023	2.39(3)
**Ce07-O016**	2.36(2)	Ce0F-O02N	2.42(3)
**Ce07-O01A**	2.35(2)	Ce0F-C03C	2.96(4)
**Ce07-O01K**	2.61(2)	Ce0G-O00N	2.508(15)
**Ce08-Ce09**	3.794(2)	Ce0G-O00U	2.10(2)
**Ce08-Ce0B**	3.826(2)	Ce0G-O010	2.116(16)
**Ce08-O00O**	2.230(16)	Ce0G-O01I	2.54(3)
**Ce08-O00X**	2.452(18)	Ce0G-O01O	2.59(2)
**Ce08-O011**	2.533(19)	Ce0G-O01R	2.38(2)
**Ce08-O01L**	2.177(18)	Ce0G-O01S	2.46(3)
**Ce08-O01Q**	2.55(2)	Ce0G-O01X	2.39(3)
**Ce08-O01U**	2.40(3)	Ce0G-O02L	2.66(3)

**Ce0G-C44**	3.07(4)
